# Methods for Quantifying Optic Disc Volume and Peripapillary Deflection Volume Using Radial Optical Coherence Tomography Scans and Association With Intracranial Pressure

**DOI:** 10.3389/fneur.2019.00798

**Published:** 2019-07-24

**Authors:** Megh Dipak Patel, Kiran Malhotra, Zainab Shirazi, Heather E. Moss

**Affiliations:** ^1^Department of Ophthalmology, Stanford University, Palo Alto, CA, United States; ^2^Department of Ophthalmology and Visual Sciences, University of Illinois College of Medicine, University of Illinois at Chicago, Chicago, IL, United States; ^3^Department of Neurology and Neurological Sciences, Stanford University, Stanford, CA, United States

**Keywords:** intracranial pressure, optic nerve, optical coherance tomography, papilledema (MeSH), idiopathic intracranial hypertension, pseudotumor cerebri

## Abstract

**Purpose:** Papilledema and peripapillary deformation of Bruch's membrane (BM) are associated with elevated intracranial pressure (ICP). We have developed a novel methodology to measure these parameters using a radial optical coherence tomography (OCT) scan pattern and apply this to test the hypothesis that ICP is associated with volumetric features of ophthalmic structures.

**Methods:** 6-radial OCT B-scans centered over the optic nerve head were acquired in 17 subjects (30 eyes) before lumbar puncture with measurement of ICP (range: 10–55 cm H_2_O). Internal limiting membrane (ILM) and BM were segmented. Three definitions of BM were studied to account for imaging artifact affecting peripapillary BM: connecting rater-identified BM margins(traditional), connecting rater-identified BM 1.6 mm on either side of the ONH(estimated), and excluding BM in the central 3.2 mm of the images(excluded). Optic nerve head volume (ONHV), BM displacement volume (BMDV) and cup volume (CV) were calculated by interpolating between B-scans. Ganglion cell complex volume (GCCV) was measured in the macula. Linear generalized estimating equations (GEE) modeled ONVH, BMDV, and CV as a function of ICP and GCCV.

**Results:** Increased ONHV was associated with elevated ICP for traditional (*p* = 0.006), estimated (*p* = 0.003) and excluded (*p* = 0.05) BM definitions. Decreased BMDV was associated with elevated ICP for traditional (*p* < 0.0005), estimated (*p* < 0.0005) and excluded (*p* = 0.001) definitions. Decreased ONHV was independently associated with decreased GCCV (*p* = 0.001) and decreased ICP (*p* = 0.031) in multivariable models. CV was neither associated with ICP nor GCCV in univariate or multivariable models.

**Conclusions:** Elevated ICP is associated with ONHV increase and BMDV decrease, calculated from OCT images accounting for image artifact. Ganglion cell atrophy affects the relationship between ICP and ONHV. OCT derived volumetric measures of the posterior eye may have application as biomarkers for elevated ICP.

## Introduction

Elevated intracranial pressure (ICP) due to idiopathic intracranial hypertension (IIH), venous sinus thrombosis, brain tumors, or other causes, results in elevated pressure of cerebrospinal fluid (CSF) in the optic nerve sheath which has multiple effects on the optic nerve and structures near it if left untreated. These include swelling of the optic nerve head due to axoplasmic flow stasis, which is an important diagnostic sign for increased ICP, (1) globe flattening, peripapillary deformation and expansion of the optic nerve sheath, all presumably due to mechanical pressure of the CSF in the nerve sheath. Ophthalmic and neuro-images of the optic nerve and surrounding globe have demonstrated differences in optic nerve head volume (ONHV), globe flattening, and peripapillary Bruch's membrane displacement (pBMd) between high and normal ICP states, and, on this basis they are candidates as diagnostic markers of high ICP states (2–5).

An important technical issue regarding the application of optical coherence tomography (OCT) derived ophthalmic structural measures as markers of ICP relates to significantly reduced BM image quality in the peripapillary region on OCT due to limited laser penetration through swollen optic nerve heads (5–7). This leads to challenges in correctly identifying and segmenting BM. Excluding the region prone to artifact from analysis has been demonstrated to have minimal impact on the relationship between ICP and two dimensional pBM shape (6). However, the use of two dimensional analysis to represent this three dimensional structure in high ICP states may not be appropriate since pBM is not radially symmetric and two dimensional scan angle impacts the ICP-pBM shape relationship (6, 8). To address this we have developed a method for three dimensional ONHV and pBMDV using OCT B-scans acquired in a radial pattern. Furthermore, we have developed strategy for addressing pBM segmentation uncertainty, which is to estimate it using image information in which there is more confidence.

We apply our novel image analysis methodology for measurement of three-dimensional optic nerve head parameters to a previously collected data set to investigate two hypotheses regarding the effect of ICP on the eye. The first hypothesis is that there are linear associations between ICP, ONHV, and Bruch's membrane displacement volume (BMDV). This relationship has been demonstrated for two dimensional pBM shape, assessed using geometric morphometric analysis, but not for three dimensional volumetric parameters (9). The second hypothesis is that ganglion cell complex (GCC) volume, a measure of ganglion cell atrophy on OCT, is a relevant covariate in the ONHV-ICP relationship. This follows from the clinical observation that retinal ganglion cell death limits the ability of their axons to manifest axoplasmic stasis responsible for ONHV increases.

## Materials and Methods

### Subjects

Patients scheduled to undergo an LP for clinical reasons were recruited from the University of Illinois at Chicago ophthalmology and neurology clinics between November 2014 and March 2016 to participate in a study of ophthalmic manifestations of high ICP, other results of which have been reported separately (6, 9, 10). The research adhered to the tenets of the Declaration of Helsinki and was approved by the University of Illinois at Chicago Institutional Review Board. Written informed consent was collected from all participants in this study after they were notified of the nature and potential outcomes and consequences of the study. Reason for LP as well as the positioning for the procedure, age, gender, and disc appearance were acquired from the medical record. Intracranial pressure was quantitatively measured during the LP as the height of the column of spinal fluid above the spinal canal using a manometer prior to removal of cerebrospinal fluid.

A total of 35 patients receiving lumbar puncture for clinical indications agreed to participate in this study. Fifteen subjects were excluded from further analysis in this study either because a successful LP was not performed or ICP was not measured. Ten eyes (2 eyes in 3 subjects and 1 eye in 4 subjects) were excluded from further analysis due to incomplete optic nerve head imaging on 1 or more B-scans. Thirty eyes from 17 participants were included in the analysis for this study. The ICP range was 10–55 cm H_2_O with an average of 28.1 cm H_2_O. LP was performed for dementia (4 participants), neuro-inflammation/MS (2 participants) and possible IIH (11 participants). Inclusion of patients with neurological disease other than high ICP was necessary to sample a range of ICP values. Low ICP (ICP ≤ 20 cm H_2_O) was seen in five participants, borderline ICP (20 ≤ ICP <25 cm H_2_O) was seen in three participants, and high ICP (ICP ≥ 25 cm H_2_O) was seen in nine participants (11).

### Image Acquisition

**Figure 1:**
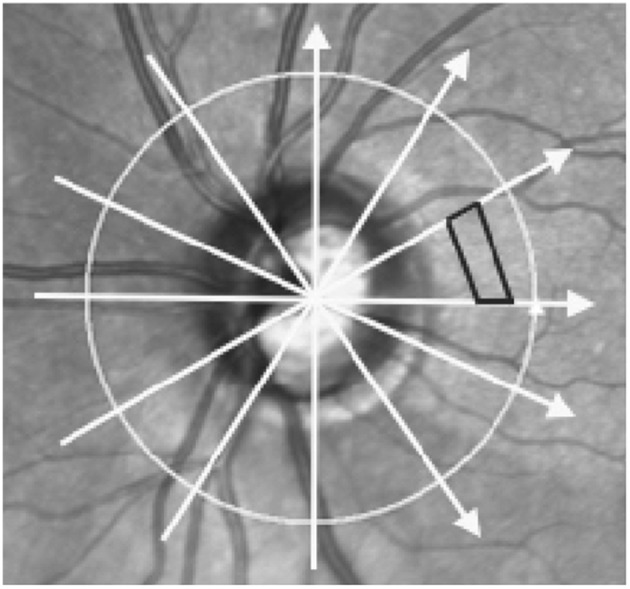
Scan pattern used to acquire optic nerve head images and method of volume calculation. Image shows a scanning Laser Ophthalmoscopy image of the optic nerve head and surrounding areas. Arrows represent the scan locations of the 6 radial optical coherence tomography (OCT) B-scans centered on the optic nerve head. Black trapezoid demonstrates how volume was interpolated between adjacent B-scans. Each volume was the sum of 510 trapezoidal prisms calculated in each of the 12 wedges formed by intersecting B-scans.

Cross-sectional OCT images of the optic nerve heads were obtained within 1 h prior to LP (Spectralis; Heidelberg Engineering, Inc., Heidelberg, Germany). The optic nerve imaging protocol consisted of six high resolution non-EDI radial B-scans separated by 30°, centered over the optic nerve (Figure 1). The scan length of each B-scan was 20° (1,024 pixels). Eyes for which any of the 6 scans truncated the vitreal surface of the optic nerve were excluded from further study. OCT scans of the macula, consisting of 19 high resolution parallel B-scans covering 20° in length and 15° in width and OCT circle scans centered on the optic nerve were also obtained prior to LP. Average retinal nerve fiber layer (RNFL) thickness was extracted for this latter scan.

### Image Analysis—Optic Nerve Scans

Raw format optic nerve OCT images (^*^.vol) were segmented using custom MATLAB-based segmentation software (A. Raza, X. Zhang, Columbia University, New York, New York) (12). Two raters independently segmented the internal limiting membrane (ILM) and BM, including identification of the opening margins surrounding the optic nerve head on each of the 6 B-scans for each eye, adjusting contrast and brightness as needed to enhance visualization. After segmenting each scan individually, inter-rater differences >2 pixels were reviewed prior to independent re-segmentation of areas with disagreement.

**Figure 2:**
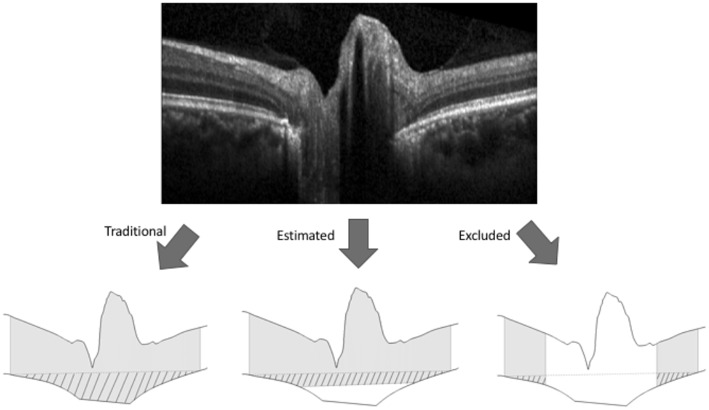
Methods for calculating volume boundaries on OCT B-scans of the optic nerve head. The internal limiting membrane and Bruch's membrane (BM) were segmented on each OCT B-scan (top image) to generate the superior and inferior boundaries (bottom images). For each of the three methods of BM representation shown in the bottom image, gray shading represents optic nerve head tissue and pattern represents BM displacement between an arbitrary secant line (dashed) and BM where the secant line was made by connecting the outermost points of BM. For the traditional method of BM representation (bottom left) the BM boundary between the rater identified margins was interpolated linearly. For the estimating representation (bottom center), the BM boundary was estimated by interpolating a line between BM points 1.6 mm on either side of the center of the scan (i.e., outside the region of segmenting uncertainty). For the excluding representation (bottom right), the central 3.2 mm of the scans were excluded from volume calculations.

Additional customized MATLAB software developed for this project was used to calculate ONHV, BMDV, and CV. The coordinates of the ILM and BM segmentations on 6 radial OCT B-scans in a single eye were scaled according to image OCT acquisition data (i.e., microns/pixel in each axis) and used as inputs. All B-scans were horizontally truncated to 5.59 mm in width, which was determined by the shortest scan in the sample. The two portions of BM on each slice (i.e., on either side of the optic nerve opening) were joined together by interpolating a straight line between the opening margins to create a continuous curve. On each B-scan the height of the optic nerve as a function of the distance from the optic nerve center was defined by the difference between the ILM and BM. Any locations with ILM dipping below the BM opening interpolation (i.e., optic nerve cup) were excluded from calculations. Trapezoidal prisms were interpolated to fill each of the 12 sectors created by the 6 radial B-scans scans to convert optic nerve height measurements into volume measurements (Figure 1). ONHV was defined as the sum of the trapezoidal prisms. BMDV was defined as the volume between an arbitrary secant plane and BM. To calculate this a secant line was defined on each B-scan to be that connecting the outermost BM points (Figure 2). BMDV calculation was done using the same methodology as for ONHV, except using the secant line instead of the ILM and including BM deflection above the secant line as negative contributions to volume. Thus, smaller and negative BMDV values correspond to more flattening of the peripapillary region into the vitreous.

Following initial image segmentation, maximum inter-rater difference in the z (depth) axis was 31 pixels for BM, with most disagreement occurring in the peripapillary region. Following consensus review maximum difference was 13 pixels. Excluding the uncertain area below the optic nerve head, maximum inter-rater difference was 6 pixels both before and after consensus review. The resulting average inter-rater difference in calculated ONHV was 0.9% (range 0–4.5%).

Cup volume (CV) was defined as the negative space created by the dip in the ILM at the center of the optic nerve. The top of the cup on each B-scan was defined by a line connecting the ILM points immediately above BM opening margins. CV was calculated as the volume between this line and the ILM. In regions where the ILM extended above the cup top line, the cup height was set to zero.

To address segmentation uncertainty regarding BM below the optic nerve head, two alternative representations of BM were used to calculate ONHV and BMDV. Both were based on the maximum distance between BM opening margins found among all scans in the sample (3.2 mm). The *estimating* technique defined BM outside the 3.2 mm diameter area according to rater segmentation and BM inside the 3.2 mm diameter area by interpolating a line on each scan to join the BM points 1.6 mm to either side of the optic nerve head center. ONHV and BMDV calculations proceeded as described above. The *excluding* technique excluded central image regions from volume calculations (Figure 2).

### Image Analysis: Macula Scans

The ILM and the inner plexiform layer/inner nuclear layer boundary were segmented automatically and corrected manually (Eye Explorer, Heidelberg Engineering Inc.). Ganglion cell complex volume (GCCV) was calculated as the volumetric difference between these layers in a 3 mm diameter circle centered on the fovea.

### Statistical Analysis

Linear generalized estimating equations (GEE) were used to construct models with each volume measure as the outcome and ICP and/or GCC as predictors. All variables were modeled as continuous variables. GEE models account for within-subject correlation and accommodate two eyes coming from a single patient in the sample. The models estimate linear coefficients for the independent variables. ONHV and BMDV were modeled using each of the BM representations (traditional, estimating, excluding). Univariable analyses were repeated using one eye per subject (right eyes, unless only one eye available) and linear regression to calculate Pearson correlation coeffients. Correlations between ONHV and BMDV were calculated using Pearson's rho for each calculation technique using one eye per subject. A similar analysis was done for ONHV and peripapillary average RNFL. Analyses were performed using SPSS V25 (IBM Inc.).

## Results

**Figure 3:**
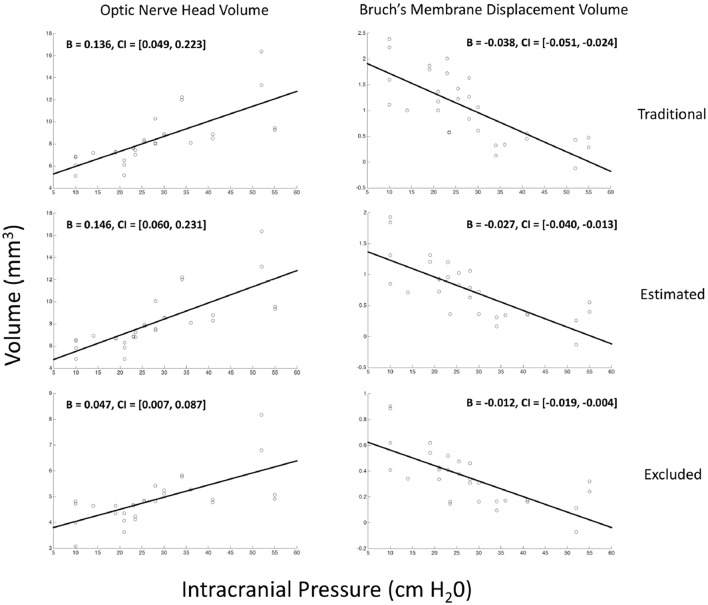
Relationship between volumetric measurements of the optic nerve head and Bruch's membrane displacement with intracranial pressure. Each marker on each scatter plot represents an eye. Y-axis is either the optic nerve head volume (ONHV) (left column) or Bruch's membrane displacement volume) calculated from OCT scans of the optic nerve head using either traditional (top row), estimated (middle row) or excluded (bottom row) representations of BM (see Figure 2). X-axis for all plots is ICP measured as opening pressure during lumbar puncture performed within 1 h after OCT image acquisition. Lines are the linear relationship between variables calculated using univariate generalized estimating equation models (see Table 1).

Thirty eyes in 17 subjects with ICP ranging from 10 to 55 cm H_2_O were included in the analysis. The ICP of subjects with both eyes included did not differ from those with one or both eyes excluded (*p* = 0.25, *t*-test). In univariate GEE models (Table 1, Figure 3), there was a linear relationship between ICP and ONHV measured using the traditional (*p* = 0.002), estimating (*p* = 0.001) and excluding (*p* = 0.022) representations of BM with the ONHV increasing with increasing ICP. There was also a linear association between ICP and BMDV measured using the traditional (*p* < 0.0005), estimating (*p* < 0.0005) and excluding (*p* = 0.002) representations of BM with BMDV decreasing with increasing ICP. Linear regression models using one eye per subject showed similar results (Table 2). ONHV and BMDV were moderately correlated for all techniques (Pearson's rho −0.56 traditional, −0.62 estimating, −0.50 excluding, *p* < 0.05 for all). ONHV was highly correlated with RNFL for all techniques (Pearson correlation coefficient (R) 0.96 traditional, 0.96 estimating, 0.88 excluding, *p* < 0.0005 for all).

**Table 1:** Univariable linear relationships between volumetric ophthalmic measurements and intracranial pressure using generalized estimating equation models (all eyes included).

BM representation^*^	**ONHV Intercept** **(mm^**3**^)** **(95% CI)**	***p***	**ONHV Slope** **(mm^**3**^/cm H_**2**_O)** **(95% CI)**	***p***
Traditional	4.606 (2.648, 6.565)	<0.001	0.136 (0.049, 0.223)	0.002
Estimated	4.066 (2.141, 5.992)	<0.001	0.146 (0.060, 0.231)	0.001
Excluded	3.570 (2.607, 4.534)	<0.001	0.047 (0.007, 0.087)	0.002
**BM representation**	**BMDV Intercept** **(mm**^**3**^**)** **(95% CI)**	***p***	**BMDV Slope** **(mm**^**3**^**/cm H**_**2**_**O)** **(95% CI)**	***p***
Traditional	2.100 (1.602, 2.597)	<0.001	−0.038 (−0.051, −0.024)	<0.001
Estimated	1.501 (1.053, 1.949)	<0.001	−0.027 (−0.040, −0.013)	<0.001
Excluded	0.683 (0.455, 0.910)	<0.001	−0.012 (−0.019, −0.004)	0.002

* *See Figure 2. BM: Bruch's membrane; ONHV, optic nerve head volume; BMDV, Bruchs membrane displacement volume*.

**Table 2:** Univariable linear relationships between volumetric ophthalmic measurements and intracranial pressure using linear models (1 eye per subject).

BM representation^*^	**ONHV Slope** **(mm^**3**^/cm H_**2**_O)** **(95% CI)**	***p***	***r***	**BMDV Slope** **(mm^**3**^/cm H_**2**_O)** **(95% CI)**	***p***	***r***
Traditional	0.123 (0.059, 0.187)	0.001	0.725	−0.035 −0.053, −0.017)	0.001	−0.732
Estimated	0.131 (0.067, 0.196)	0.001	0.746	−0.025 (−0.038, −0.012)	0.001	−0.717
Excluded	0.045 (0.019, 0.070)	0.002	0.694	−0.011 (−0.017, −0.005)	0.002	−0.690

* *See Figure 2. BM, Bruch's membrane; ONHV, optic nerve head volume; BMDV, Bruchs membrane displacement volume*.

**Figure 4:**
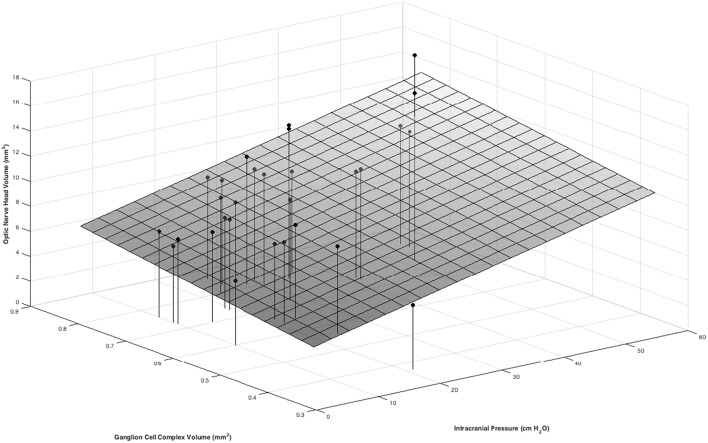
Positive associations between ONHV and both ICP and GCC volume in a multivariate model. ICP was measured as the opening pressure during lumbar puncture done within 1 h after OCT image acquisition. Macula GCC volume is tissue between the ILM and the inner plexiform layer/ inner nuclear layer boundary in a 3 mm diameter circle centered on the fovea. ONHV measurements were calculated using the traditional representation (see Figure 2).

In multivariable models ONHV was independently associated with both ICP (*p* = 0.014) and GCC volume (*p* = 0.002) using the traditional representation of BM (Table 3, Figure 4). When adjusting for GCC, ONHV remained linearly associated with ICP when using the estimating BM representation (*p* = 0.006) but not when using the excluding BM representation (*p* = 0.086). BMDV was not associated with GCC in multivariable models. Pearson correlation coefficient for multivariable models with one eye per subject were 0.801 (traditional), 0.811 (estimating), 0.805 (excluding).

**Table 3:** Multivariable linear generalized estimating equation models of optic nerve head volume as a function of intracranial pressure and macula ganglion cell complex volume.

BM representation^*^	**Intercept** **(mm^**3**^)** **(95% CI)**	***p***	**ICP coefficient** **(mm^**3**^/cm H_**2**_O)** **(95% CI)**	***p***	**GCC coefficient** **(mm^**3**^/ mm^**3**^)** **(95% CI)**	***p***
Traditional	0.756 (−1.712, 3.223)	0.548	0.118 (0.024, 0.221)	0.014	5.930 (2.194, 9.666)	0.002
Estimated	0.430 (−2.150, 3.010)	0.744	0.128 (0.036, 0.220)	0.006	5.600 (1.575, 9.624)	0.006
Excluded	1.703 (0.737, 2.668)	0.001	0.038 (−0.005, 0.081)	0.086	2.876 (1.386, 4.366)	<0.001

* *See Figure 2. BM, Bruch's membrane; ONHV, optic nerve head volume; ICP, intracranial pressure; GCC, macula ganglion cell complex volume*.

In univariate models, CV was not associated with ICP (−0.003 mm^3^/cm H_2_O; 95% CI−0.10, 0.004; *p* = 405). In multivariable models, CV was associated with neither ICP (−0.002 mm^3^/cm H_2_O; 95% CI −0.010, 0.0.006, *p* = 0.666) nor GCC (−0.365 mm^3^/mm^3^; p95% CI −1.065, 0.335; *p* = 0.307).

## Discussion

In this study, a novel methodology for volumetric calculations of optic nerve head tissue, peripapillary deformation and cup volume were compared against ICP. The results support our hypotheses of a quantitative relationship between ONHV and BMDV and ICP, but not of CV and ICP, which has not been previously reported, and demonstrate macular GCC thickness to be a relevant co-variate in the ONHV-ICP relationship. Our findings contribute to the growing body of literature regarding methodology for OCT image analysis of edematous optic nerve heads and the relationship between ICP and structural ophthalmic measures.

A significant challenge in calculating optic nerve shape or volume metrics related to ICP using OCT is the impact of swollen optic nerves on accurate identification of Bruch's membrane (5–7). It has been proposed that Bruch's membrane opening widening associated with high ICP states may be artifactual related to poor identification of BM margins (5, 13). This was the region prone to greatest disagreement between independent raters in our study. The current study explores two different methods of addressing the uncertainty in segmenting BM by estimating and excluding regions of the membrane to increase reliability in volume measurements. Results indicate that the BMDV-ICP relationship is similar across techniques. However, the ONHV relationship is less robust using the excluding technique, which is not surprising since this excludes the region of the image that represents that pathology being measured. Our findings suggest that using volumetric measurements to describe structural changes based on estimating techniques for imaging regions prone to uncertainty in segmentation is a reasonable approach that will reduce the impact of image artifact. The tight correlation between ONHV and RNFL, suggest that RNFL, for which robust automated segmentation algorithms are available on commercial OCT devices may be an appropriate ONHV surrogate. While other imaging modalities such as MRI are not prone to image artifact in this region, their resolution is substantially less and their acquisition substantially more burdensome.

Prior studies of optic nerve shape and volume have used volume OCT scans (14, 15) whereas the present study utilizes 6 radial scans centered over the optic nerve. This scan pattern allows for cross sectional imaging of the optic nerve head and BM opening that is near perpendicular to the BM margins. We believe this facilitates identification of the BM opening as opposed to parallel volume scans which are problematic to segment where they are nearly tangential to the BM opening. Study of denser raster scan patterns is an area for future study as is a direct comparison between volume and radial scan patterns.

ONHV is a logical measure of ophthalmic change due to elevated ICP since it is a quantification of papilledema, an important clinical sign of elevated ICP. Previous studies have shown ONHV to be associated with a qualitative categorical assessment of the optic nerve (i.e., Frisén scale) (15, 16). It is potentially superior to the Frisén scale given poor reliability of the latter metric (17). ONHV derived from OCT scans has previously been shown to decrease in size in individuals with IIH following treatment with a weight loss intervention with and without acetazolamide (5). Our results contribute evidence that the ONHV is quantitatively associated with baseline ICP. Clinically it is well-appreciated that papilledema can be impacted by ganglion cell demise since dead cells cannot swell. However, the impact of this on the quantitative ONHV-ICP relationship has not been previously reported. We find that ONHV is independently associated with both ICP and GCC volume, a marker of retinal ganglion cell and axonal volume, impacted positively by axonal swelling and negatively by tissue loss. Therefore, we propose that future studies of ONHV as a marker for ICP account for ganglion cell atrophy.

BMDV is an attractive marker of ICP since it is thought to reflect a direct mechanical result of elevated ICP on the posterior globe. Prior studies have demonstrated associations between high ICP and BM deflection into the eye using 2-D shape analysis of OCT and surface topography of MRI (6, 9, 18, 19) 3-D shape analysis applied to OCT demonstrated resolution of BM deflection within individuals undergoing ICP treatment over months (5) and 2-D shape analysis demonstrated detectable changes in hours following ICP lowering (9). Though changes in BMDV and ONHV were moderately correlated in our study, they may have distinct clinical roles as suggested by a previous longitudinal analysis demonstrating decoupling between BM shape and ONHV (5). We studied a novel volumetric measurement (BMDV) which address the concerns of using a two-dimensional scan to characterize BM based on an assumption of radial symmetry (8, 20). This methodology can be directly calculated from segmented OCT scans. We find that BMDV decreases as baseline ICP increases, which corresponds to further inward deflection of peripapillary tissues with increasing levels of ICP.

Cup area is an important clinical feature in assessment of optic nerve structure. Clinically the cup is observed to fill in in association with optic nerve head swelling of various causes. However, our results do not support use of our methodology of measuring cup volume as an imaging marker of high ICP. The lack of association may be attributed to the method in which cup volume was calculated, which relied on the ILM margins of the optic nerve to define the anterior cup boundaries. Thus, swelling of the optic nerve increased cup height by our definition which acts to increase volume estimates even if the cross-sectional area is less due to axon swelling.

This study recognizes a number of limitations in regard to the sample set and methods of data collection. The study analyzes data from a relatively small sample set to demonstrate application of novel image analysis methods. Though both eyes were used per subject in analyses, univariate results were not impacted by limiting analysis to one eye per subject. Study design leveraged LPs performed for clinical reasons in order to sample a broad range of ICPs. As a result many subjects had neurological disease. The resulting analysis assumes that the effects ICP effects on optic nerve structures are independent of other neurological diseases, which is reasonable for an initial investigation of this kind. This data set has previously been analyzed with respect to 2-dimensional BM shape on B-scans along the nasal-temporal axis, using geometric morphometric analysis, and the present analysis expands this to consider 3-dimensional volumes reflecting BM, but also ONHV and CV (6, 9, 10). This study focused on a broad range of ICP in order to define the quantitative relationships with the quantitative OCT across the a normal and high ICP range. Future work studying a larger sample of subjects with high ICP is needed to address the application of BMDV and ONHV to the clinical problem of differentiating levels of high ICP within and between subjects. Consideration of covariates known to be associated with papilledema such as optic canal diameter will be important in such a study (21).

In conclusion, we contribute a novel method of optic nerve volume calculation using radial scans through the optic nerve head and demonstrate that estimating BM in the region of the image prone to artifact to be a potential approach to address the difficulty of accurate segmentation in this region. We applied this analysis methodology to OCT images of the optic nerve head to demonstrate quantitative linear relationships between volumetric measurements of the optic nerve and ICP. These results support development of these metrics as biomarkers of ICP, which would require substantial additional study. We find that retinal ganglion cell atrophy confounds the ICP/ONHV relationship and suggest this is because cell death limits axoplasmic stasis and therefore reduces the effect of ICP on ONHV.

## Data Availability

The raw data supporting the conclusions of this manuscript will be made available by the authors, without undue reservation, to any qualified researcher.

## Ethics Statement

This study was carried out in accordance with the recommendations of the University of Illinois Office for the Protection of Research Subjects Institutional Review Board with written informed consent from all subjects. All subjects gave written informed consent in accordance with the Declaration of Helsinki. The protocol was approved by the University of Illinois Office for the Protection of Research Subjects.

## Author Contributions

MP, KM, and HM contributed to conception and design of the study. ZS collected the data and organized the database. MP wrote the first draft of the manuscript. All authors contributed to manuscript revision, read and approved the submitted version.

### Conflict of Interest Statement

The authors declare that the research was conducted in the absence of any commercial or financial relationships that could be construed as a potential conflict of interest.
